# Protocol for cluster randomized evaluation of reaching married adolescents - a gender-synchronized intervention to increase modern contraceptive use among married adolescent girls and young women and their husbands in Niger

**DOI:** 10.1186/s12978-019-0841-3

**Published:** 2019-12-18

**Authors:** Sneha Challa, Stephanie M. DeLong, Nicole Carter, Nicole Johns, Holly Shakya, Sabrina C. Boyce, Ricardo Vera-Monroy, Sani Aliou, Fatouma A. Ibrahima, Mohamad I. Brooks, Caitlin Corneliess, Claire Moodie, Abdoul Moumouni Nouhou, Illa Souley, Anita Raj, Jay G. Silverman

**Affiliations:** 1 Center on Gender Equity and Health, University of California San Diego, San Diego, USA; 2 Pathfinder International, Boston, USA; 3 The OASIS Initiative, Niamey, Niger

**Keywords:** Global health, Contraception, Sub-Saharan Africa, Adolescents

## Abstract

**Background:**

Early marriage and early childbearing are highly prevalent in Niger with 75% of girls married before age 18 years and 42% of girls giving birth between ages 15 and 18 years. In 2012, only 7% of all 15–19-year-old married adolescents (male and female) reported use of a modern contraceptive method with barriers including misinformation, and social norms unsupportive of contraception. To meet the needs of married adolescents and their husbands in Niger, the Reaching Married Adolescents (RMA) program was developed with the goal of improving modern contraceptive method uptake in the Dosso region of Niger.

**Methods:**

Using a four-arm cluster randomized control design, the RMA study seeks to assess whether household visits only (Arm 1), small group discussions only (Arm 2), or a combination of both (Arm 3), as compared to controls (no intervention – Arm 4), improve modern contraceptive method use among married adolescent girls and young women (AGYW), age 13–19 years-old, in three districts of the Dosso region. Intervention conditions were randomly assigned across the three districts, Dosso, Doutchi, and Loga. Within each district, eligible villages were assigned to either that intervention condition or to the control condition (12 intervention and 4 control per district). Across the three intervention conditions, community dialogues regarding modern contraceptive use were also implemented. Data for the study was collected at baseline (April – June 2016), at 24 months post-intervention (April – June 2018), and a final round of data collection will occur at 40 months post-intervention (October – December 2019).

**Discussion:**

The RMA intervention is a gender-synchronized and community-based program implemented among married adolescent girls and their husbands in the context of rural Niger. The intervention is designed to provide education about modern contraception and to promote gender equity in order to increase uptake of modern contraceptive methods. Results from this cluster randomized control study will contribute to the knowledge base regarding the utility of male engagement as a strategy within community-level approaches to promote modern contraceptive method use in the high need context of West Africa.

**Trial registration:**

Registered October 2017 - ClinicalTrials.gov NCT03226730.

## Plain English summary

In Niger, early marriage is common, but family planning is not generally practiced leading to significant early childbearing and increasing the health risks for young married girls. To meet the needs of these young married girls, the Reaching Married Adolescents (RMA) program was designed with the goal of improving use of modern contraceptive methods. Three districts of the Dosso region of Niger were randomly assigned to receive one of three intervention approaches: 1) small group discussions, to strengthen social bonds, 2) household visits, to provide knowledge about reproductive health and family planning, or 3) a combination of small group discussions and household visits. From these three districts, 48 villages were randomly selected (12 participating in the intervention approach assigned to the respective district and 4 to serve as a control). Villages that were selected to participate in the intervention also participated in community dialogue sessions to create an environment supportive of family planning and healthy timing and spacing of pregnancy. From each village, 25 married adolescent girls (ages 13–19) and their husbands were randomly selected to participate. Data for the study was collected at pre-intervention implementation (April – June 2016), at 24 months post-intervention implementation (April – June 2018), and a final round of data collection will occur at 40 months post-intervention implementation (October – December 2019). Results from this study will contribute to the knowledge base regarding the utility of involving men as a strategy within community-based programs to promote modern contraceptive method use in the high need context of West Africa.

## Background

Recent Demographic and Health Survey (DHS) data from Niger show that 76% of girls marry before the age of 18 and 28% before the age of 15 [1]. Though marrying under age 18 years is permitted by law (legal age of marriage is 15 years for girls) [2], child marriage is associated with poor adolescent health and development, including neonatal and maternal mortality, lack of formal education, and intimate partner violence (IPV). Child marriage is also associated with early childbearing and high adolescent fertility in Niger and multiple other contexts [3–5]. Approximately 9% of girls give birth to a child before age 15 years and 42% give birth between ages 15 and 18 years in this West African nation, [6] with adolescent and total fertility in Niger higher than any country globally [7].

Girls who marry at younger ages in Niger are less likely than those marrying at older ages to utilize maternal health care services (e.g., antenatal visits and institutional delivery), contributing to poor health outcomes [8]. In 2012 in Niger, only 7% of all 15–19 year-old married adolescents (male and female) reported use of a modern contraceptive method [9]. Barriers to modern contraceptive use include lack of access, lack of awareness, misinformation and lack of knowledge, and opposition to use by partners and family [9]. Multiple social norms (i.e., shared social expectations) have been documented in this and other sub-Saharan cultural contexts that additionally impede modern contraceptive uptake [10]. Programs designed to combat early and frequent childbearing in these regions by promoting modern contraceptive use face the challenge of addressing these many barriers.

To meet the needs of married adolescents and their husbands in Niger, Pathfinder International (Pathfinder) developed an intervention aimed at improving modern contraceptive method uptake in the Dosso region of Niger. The Reaching Married Adolescents (RMA) program focuses on increasing knowledge of modern contraceptive methods and changing relevant attitudes and norms that may impede use via household visits and small group discussions with married adolescent girls and young women (AGYW) and their husbands. Complementing the focus on contraceptive use, this gender-synchronized approach also engages men and women to challenge gender norms and adopt attitudes supportive of gender equity, particularly with respect to female autonomy and decision-making regarding sexual and reproductive health (SRH). Researchers from the Center on Gender Equity and Health at the University of California, San Diego (UCSD) [11] are carrying out a cluster randomized control trial of this intervention to assess its impact on both modern contraceptive use and relevant gender equity outcomes.

### Study overview and design

The purpose of the RMA evaluation study is to assess the efficacy of the RMA intervention using a four-arm cluster randomized control design (October 7, 2017, retrospectively registered, https://clinicaltrials.gov/ct2/show/NCT03226730, Clinical Trials ID: NCT03226730). Specifically, this study seeks to assess whether household visits only (Arm 1), small group discussions only (Arm 2), or a combination of both (Arm 3), as compared to controls (no intervention – Arm 4), improve modern contraceptive method use among married AGYW, age 13–19 years-old, in three districts of the Dosso region of Niger. Based on our conceptual model, we hypothesize that married AGYW that participate in any of the three intervention arms will report: 1) improvement in knowledge of modern contraceptive methods, 2) attitudes more supportive of contraceptive use and gender equity, 3) perceived norms within their communities as more supportive of contraceptive use and gender equity, 4) increased self-efficacy to use a contraceptive method, 5) intention to use a modern contraceptive method, 6) reduction in IPV, and 7) increased use of modern contraceptive methods. Secondarily, reflective of the male engagement elements of RMA, we hypothesize that higher levels of participation of husbands of married AGYW will be associated with greater increases in modern contraceptive use and greater reductions in intimate partner violence, relative to lower levels of husband participation or no participation at all.

**Fig. 1:**
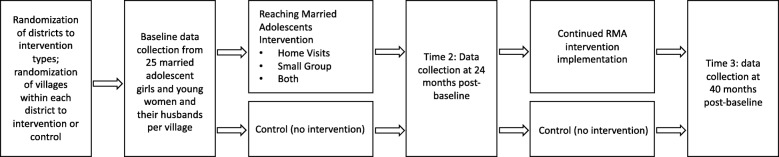
Study Design

Quantitative baseline data (pre-intervention) were collected from April–June 2016 using structured surveys. These same data are collected at 24 months post-baseline (Time 2) from April – June 2018, and 40 months post-baseline (Time 3) from October – December 2019 (see Fig. 1).

#### Conceptual model

**Fig. 2:**
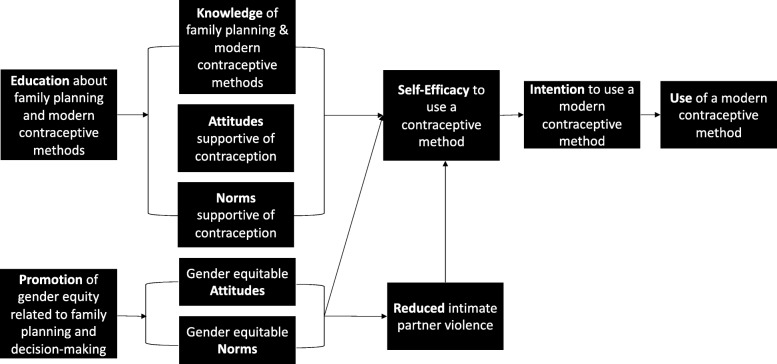
Conceptual Model of the RMA Intervention

Adapted from Fishbein and Yzer’s modified Theory of Reasoned Action, [12] Fig. 2 depicts the conceptual model of the RMA intervention and the hypothesized pathways through which the intervention promotes change. The primary goal of the RMA intervention is to increase use of modern contraceptive methods among married adolescents age 13–19 years-old and their husbands in Niger. The RMA intervention provides education and counseling on use of modern contraceptive methods and the importance of delaying and spacing pregnancies, particularly for adolescents, alongside promotion of gender equitable attitudes towards, and norms regarding joint decision-making and use of modern contraception. Education on modern contraceptive methods, inclusive of reproductive anatomy and dispelling common misconceptions regarding contraceptives and fertility, is hypothesized to improve knowledge of, attitudes towards, and norms regarding contraceptive use. In parallel, promotion of gender equitable attitudes and norms is hypothesized to reduce perpetration of IPV. Research has demonstrated that IPV is associated with self-efficacy around SRH [13] thus, together, these elements are hypothesized to increase self-efficacy to use modern contraceptive methods. This in turn, will increase intention to use a modern contraceptive method finally resulting in actual modern contraceptive method use. Modern contraceptive methods promoted include intrauterine devices (IUD), injectable contraceptives, contraceptive implants, oral contraceptive pills, male condoms, female condoms, emergency contraception, and lactational amenorrhea (LAM).

#### District randomization, village selection, and randomization

**Fig. 3:**
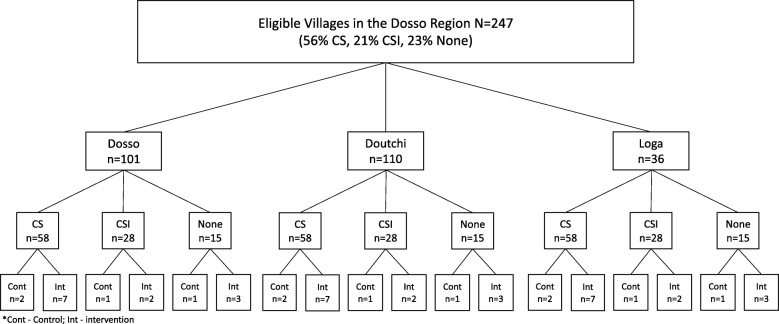
Selection and Randomization Procedures

The Ministry of Health chose the Dosso region of Niger for the RMA intervention. This region has five districts from which three districts, Dosso, Doutchi, and Loga, were selected for participation. Based on the logistical challenges of providing all RMA intervention approaches in each district, each district was randomly assigned to one of the three intervention approaches (home visits alone, small groups alone, or a combination), with controls selected across all three districts. Based on power analyses (described below), the minimum number of clusters in each of the four arms was 12, requiring inclusion of 16 villages across each of the three districts (12 intervention and 4 control). Village selection and randomization followed a multi-stage process whereby eligible villages in the three districts were first identified based on the following criteria: 1) rural (i.e., not designated as a market town or city), 2) Hausa- or Zarma-speaking, 3) a minimum of 1000 inhabitants, and 4) located within 5 km of a *centre de santé intégré* (CSI) or *case de santé* (CS) health facility. There were 101 villages in Dosso, 110 in Doutchi, and 36 in Loga that met these criteria. From the eligible villages, we then stratified villages based on three level of health service provision: 1) villages with CSIs, larger health facilities serving multiple villages where doctors, nurses, and midwives provide the full contraceptive method mix and related counseling; 2) villages with CSs, village-level facilities where community health agents provide a subset of contraceptive methods; and 3) villages with neither CSI nor CS. Among the 247 total eligible villages across the three districts, 21% had a CSI, 56% had a CS, and 23% had neither. In order to select a sample of villages within each district that was representative of the distribution of health facilities across the region, we randomly selected nine villages with a CS, three with a CSI, and four with neither in each of the three districts. To create a control arm representative of villages in each district, and that included representation of all levels of health facility, we randomly assigned four of the total 16 villages per district to the control condition, consisting of two villages with a CS, 1 with a CSI, and 1 with neither. The remaining 12 villages were assigned to the intervention condition assigned to that district (see Fig. 3).

##### Additional randomization for the 40-month follow-up

Prior to the 40-month follow up, six intervention villages per district will be randomly assigned to continue to receive supervision from Nurse Supervisors while the remaining six intervention villages in each district will continue intervention activities with no supervision. Pairwise (block) randomization within each intervention arm by current modern contraceptive use rate will be conducted to ensure similar distributions of modern contraceptive utilization in those villages continuing vs ceasing supervision. All four control villages in each district will continue to serve as controls. This additional randomization will allow us to determine sustainability of the effects of the intervention without direct supervision.

#### Power

Power calculations for our study were conducted a priori based on our primary outcome of interest, modern contraceptive method use (reported by the adolescent wives in our sample). We used pairwise tests for time by treatment effects comparing each intervention group individually to the control group. We chose to account for a moderate intra-cluster correlation coefficient – kappa = 0.05 and an attrition rate of 10%. We also chose to account for the ability to detect an effect size of 2.0 greater odds of contraceptive method use, based on literature reviews of evaluations of interventions seeking to improve contraceptive use, as well as our own experience conducting such trials in other regions. Four arms with 12 clusters nested within each arm, and a sample size of 240 couples per arm (300 couples at baseline with 20% expected attrition; 1200 total baseline participating couples) was calculated to provide 80% power to detect associations of this magnitude.

#### Participants – recruitment, eligibility, consent, and ethics

##### Recruitment and eligibility

At baseline, participants were selected for recruitment based on a complete listing of married AGYW and their husbands provided by leaders in each of the participating villages. A random numeric sequence was attached to the listing and those couples numbered 1–25 were selected for recruitment. These households were then approached by trained male and female research assistants (RAs) fluent in both French and Hausa and/or Zarma accompanied by a village guide (appointed by the village leaders) to determine interest in participating in the study. If more than one adolescent wife from a household was randomly selected for inclusion, we included only the youngest eligible wife in the study. In accordance with cultural norms, village guides and RAs first approached the heads of household (if the husbands were not the head of households) in order to receive permission to approach other household members.

To confirm eligibility at baseline, RAs asked the head of household to provide a household roster with relationships and ages. Age was then reconfirmed via surveys with individual AGYW and husbands. At all timepoints, up to three attempts are made to reach the selected participants. At baseline, if the person could not be reached after these three attempts, a replacement was chosen from the original listing based on assignment of the next number in the sequence (i.e., 26, and then 27 if required, etc.). This process continued until 25 eligible couples were enrolled in each village.

At baseline, couples were recruited based on the eligibility of the adolescent wife. Adolescent girls and young women were eligible based on the following criteria: 1) age 13–19 years, 2) married, 3) fluent in Hausa or Zarma (local languages), 4) residing in the village with no plans to move out of the village in next 18 months or to be away from the village for more than 6 months during that period, 5) not currently sterilized (to enable measurement of changes in use of reversible methods of contraception from baseline to follow-up), and 6) providing informed consent.

##### Consent and ethics

After eligibility is confirmed based on data provided by the head of household, the husband of the AGYW is approached to ensure that there are no objections to his wife’s participation in the study; this is done to conform to local cultural norms and to minimize risk. If either the head of household or husband does not assent to participation of both the wife and husband, the couple is determined to be ineligible. A gender-matched RA then accompanies each member of eligible couples to a private location of their choosing to obtain consent and conduct the survey. Verbal consent is assessed after describing the study and participation requirements in detail. In keeping with the World Health Organization guidelines for ethical conduct on research on violence against women [14, 15]. precautions were taken to ensure the safety of adolescent female participants, as well as their privacy, and the confidentiality of their responses. To ensure that married AGYW do not feel pressured or coerced to participate based on husband assent, RAs assure them that consent is truly voluntary and that they are free to decline to participate. Further, only one woman per household is recruited for participation, and questions about experiences of violence were not asked of husbands, in order to minimize any potential risk to her safety arising from her participation. If during the course of the interview, a married AGYW reports IPV, she is offered the option for confidential psychological support to increase her safety. The Pathfinder Program Director, a woman trained in women’s health and safety, is able to provide this service. All study protocols were approved by the Institutional Review Boards (IRBs) at the University of California, San Diego (protocol number 160407S, 03/08/16) and of the Nigerien Ministry of Health. Any modifications to this protocol were reported to the both ethics committees (see Additional file 1).

### RMA intervention

#### Intervention description

The design of the RMA intervention was informed by Pathfinder’s experience implementing similar programs in India [16], Uganda [17], and Burkina Faso [18] that have also included activities involving both men and women, along with community members to promote voluntary contraceptive use.

The two primary components of the RMA intervention include: 1) household visits made by trained community health workers (*relais*), recruited from within the communities to provide education and promote uptake of modern contraceptives and 2) small group discussions held by mentors (also trained community members) to promote dialogue around delaying and spacing births, use of modern contraceptive methods, and relevant cultural and gender norms. The design of the program is based on the socio-ecological model and designed to target the multiple levels of influence on contraceptive use among married AGYW and their husbands in the context of rural Niger. These two main intervention activities are supplemented with community dialogues held in villages across all three districts assigned to any of the three intervention arms to create an enabling environment for healthy timing and spacing of pregnancy (HTSP) and contraceptive method use. Table 1 shows a brief description of all intervention components.

**Table 1:** Summary of RMA Intervention Components

	Intervention Component	Intervention Activities and Target Audience	Facilitators and Dosage	District
Primary Intervention Components	Household Visits	Home visits by *relais* are made separately to married adolescent girls and young women (AGYW) and their husbands to provide information and counseling on healthy timing and spacing of pregnancy (HTSP) and contraception.	Female and male *relais*, 12 visits, 1x/month	Loga, Dosso
	Small Group Discussions	Married AGYW – groups foster discussions focused on health and life skills, puberty, contraception, gender, violence; participants also discuss their hopes, dreams, and challenges in a way that promotes social cohesion. Husbands - groups foster reflection and dialogue to encourage more equitable gender norms, support for contraception use for HTSP, positive health seeking behavior for himself and his family, and increased couple communication.	Female and male mentors, 24 sessions for married AGYW 2x/month, 12 sessions for husbands 1x/month	Doutchi, Dosso
Supplementary Intervention Component	Community Dialogues	Community gatekeepers including traditional and community leaders, parents and in-laws participate in community dialogue and reflection sessions to create an environment supportive of HTSP and contraception use.	Four community facilitators (FACOMs) in each district, 1x/month	Loga, Dosso, Doutchi

##### Intervention component 1: household visits

Household visits are conducted by trained *relais* in the homes of married AGYW and their husbands and include provision of education regarding modern contraceptive methods to counter misinformation, promotion of acceptability of contraceptive use and of engagement with the health system to receive family planning (FP) counseling and a modern contraceptive method. *Relais* are gender-matched, i.e., a female *relais* is assigned to each married AGYW, and a male *relais* is assigned to the married AGYW’s husband. Each *relais* speaks to the assigned individual for one hour each month in the home. During these visits, *relais* use project-developed tools and an illustrated guide that supports their delivery of the twelve educational counseling sessions focused on FP counseling. At the first home visit, the *relais* meets with all interested adult family members to explain the RMA program objectives and request permission from the head of household to visit the home and conduct the RMA sessions. Households in this region of rural Niger often comprise multiple related families led by the eldest man.

*Relais* are members of the community who are committed to providing the RMA curriculum in order to help the community. Selection criteria for *relais* include: 1) age 20–45 years, 2) married 3) residing in the village, 4) availability to provide RMA year-round, 5) ability to read and write French (local languages of Hausa and Zarma are not typically written), and 6) favorable attitude towards modern contraception. *Relais* were recruited during village meetings prior to implementation of RMA. In each of the intervention villages assigned to receive household visits, a representative of the program met with the village chief and select community members to present the objectives of RMA, the elements of the intervention, and the selection criteria for *relais*. They then propose names of those that they believe best meet the selection criteria for *relais*.

Training of *relais* takes place over a seven-day period with five days of training sessions, one day for preparation, and one day for reporting. Training is conducted in collaboration with the regional public health division directors as well as health center agents. Training sessions cover modern contraceptive methods, HTSP, gender equity, and adolescent rights with a focus on how to provide education and counseling to participants around these issues.

##### Intervention component 2: small group discussions

Separate small group discussions, conducted by mentors, are held with married AGYW and their husbands. These small groups are formed in each village to foster social cohesion, to build trust, and to promote dialogue related to contraception and SRH within the community. Groups consist of 10 to 13 participants each and are hosted at a health center – twice per month for married AGYW and once per month for their husbands. A female mentor facilitates all group sessions with married AGYW, and a male mentor facilitates all group sessions with their husbands, with additional support for the groups provided by health center staff and community leaders. By collaborating with community leaders, the project hopes to transform resistance to contraception, gender inequitable attitudes, and fertility norms supportive of early and frequent childbearing.

Mentors, who lead the small group discussions, are members of the community and selected by project staff with input from other community members. Selection criteria for mentors were the same as for relais. As with *relais*, training of mentors is conducted in collaboration with the regional directors of public health as well as health center agents. The mentors’ training consists of a five-day workshop with modules on leading and facilitating group discussions about gender equity, the reproductive system, modern contraceptive methods, HTSP, and the health system/services available at each level of the health system.

##### Supplementary intervention component: community dialogues

Community dialogues supplement the two main intervention approaches, household visits and small group discussions, and are a constant in all three intervention arms. Once each month in every intervention village, all adolescent and adult members of the community gather to participate in community dialogue and reflection sessions. This activity is meant to create an environment supportive of modern contraceptive method use and HTSP. Town criers alert community members to these gatherings and village chiefs and religious leaders encourage participation. Sessions are held in the village mosque or center of the village.

Community dialogue facilitators (FACOMs) who lead these sessions - four FACOMs per district (12 in total) - are a mix of men and women selected at the district level by district-level health staff and project staff. Selection of FACOMs also includes input from village leaders. Criteria for selection were the same as for *relais* and mentors. FACOMs receive a seven-day training that, similar to the training of mentors, includes modules on leading and facilitating group discussions related to gender equity, the reproductive system, modern contraceptive methods, and HTSP.

#### Intervention monitoring

To monitor intervention implementation, Nurse Supervisors provided direct supervision of frontline workers (*relais* conducting household visits, mentors leading small group discussions, and FACOMs organizing community dialogues) as well as quarterly focus group discussions. On a monthly basis, Nurse Supervisors visit the frontline workers to ensure the implementation of intervention activities. Intervention implementation is tracked via logs maintained by all frontline workers in which they record the dates and types of intervention activities conducted. At each monthly visit, Nurse Supervisors review logs to identify gaps in implementation. Every other month, frontline workers gather with Nurse Supervisors to plan activities and themes of household visits, small group discussions, and community dialogues. During these meetings, frontline workers and Nurse Supervisors also reflect on what is working well, what is going differently than planned, and what can be improved and how to enact these improvements. At these meetings, frontline workers receive transportation fees and a per diem for their attendance.

In addition, during the first 12 months of intervention implementation, three quarterly meetings were held with a selection of frontline workers (*N* = 12) at the district level. At these meetings, a facilitator held focus group discussions to encourage reflections on challenges to faithful implementation and to collectively develop solutions to address these challenges. Transcripts of these focus groups were used to improve implementation. Focus group discussions were conducted in June 2017, October 2017, and March 2018 with *relais*, mentors and FACOMs.

#### Data collection

As described above, baseline (pre-intervention) data collection occurred from April through June 2016, to be followed by 24-month post-baseline (Time 2) data collection from April – June 2018, and 40-month post-baseline (Time 3) data collection from October – December 2019. Separate questionnaires for wives and husbands were created based on existing reliable and valid measures for low-resource settings such as those included in the Demographic and Health Surveys [19]. Survey measures include items capturing receipt of and participation in each element of the RMA intervention, as well as the primary outcome (modern contraceptive method use), secondary outcomes (knowledge of contraceptive methods, attitudes towards contraception, norms surrounding contraceptive method use, contraceptive method use self-efficacy, intention to use a modern contraceptive method, IPV), and demographics.

Quantitative surveys were first drafted in English, then professionally translated and back-translated to and from French for programming. Research Assistants (RAs), using tablet computers programmed with the survey in French, read all survey instructions and questions verbally to participants in either Hausa or Zarma, based on the preference of the participant. This on-site translation is necessary due to Hausa and Zarma being primarily spoken languages. Research Assistants received extensive training to ensure accurate and reliable translation across RAs. Survey administration takes approximately 45–60 min. Research Assistants were selected, trained and supervised by in-country research partner, L’Initiative OASIS Niger (OASIS), based on RAs previous training and experience in quantitative data collection, as well as their fluency in French and Hausa and/or Zarma. Training of RAs was conducted over five days by OASIS, and included sessions about research ethics and informed consent, procedures to minimize and assess participant distress, referrals for participants reporting IPV, use of the tablet computer to administer the quantitative survey, and data safety, storage and transfer.

Before data collection at 24 months and 40 months, additional steps are taken to ensure participant retention in the study. Staff members from both Pathfinder and OASIS confirm permission from village leaders for upcoming data collection and visit villages to make residents aware that data collection will soon begin. In the process, they confirm participants’ presence in the village and ask residents to encourage participating family/friends who may have migrated for work to return to the village for the data collection period if possible.

#### Primary outcome measure

##### Contraceptive method use

The main outcome of interest for the RMA intervention and evaluation is modern contraceptive method use as reported by the married adolescent wife participants. Current modern contraceptive use will be assessed using data from the following survey items adapted from the DHS [19]: 1) “Are you or your husband currently doing something or using any method to space or delay pregnancy?” 2) if yes, “which method are you currently using?” Thus, if a married AGYW participant responds that, yes, they or their husband are currently using a modern method (IUD, injectable, implant, pill, condom, female condom, emergency contraception, or LAM) they will be considered current modern contraceptive method users. Women pregnant at the time of the survey will be excluded in analyses of this outcome.

#### Secondary outcome measures

##### Intimate partner violence

Other outcomes of interest include physical and sexual IPV as reported by the married AGYW participant. To assess physical and sexual IPV, we have adapted items from the DHS Domestic Violence Module [19] (DHS module adapted from recommendations made by the WHO based on the Multi-country Study on Women’s Health and Violence against Women) [20]. To assess physical IPV, female participants are asked whether their husbands have done any of the following things ever and in the past 12 months: 1) pushed them, shaken them, or thrown something at them; 2) slapped them; 3) twisted their arms or pulled their hair; 4) hit them with a fist or with something that could hurt them; 5) kicked them, dragged them, or beaten them up; or 6) tried to choke them or burn them. If participants respond that they have experienced any of these forms of abuse events in the past 12 months, they are considered to have experienced recent physical IPV.

To assess sexual IPV, participants are asked whether their husbands have done any of the following things ever and in the past 12 months: 1) physically forced them to have sexual intercourse when they did not want to, or 2) physically forced them to perform any other sexual acts they did not want to. If participants respond that they have experienced either of these forms of sexual violence in the past 12 months, they are considered to have experienced recent sexual IPV.

##### Knowledge of contraceptive methods

This measure is constructed of 14 items covering knowledge of modern contraceptive methods and reproductive health. Items included in this measure were adapted from recommendations by USAID and Intrahealth International, and research by Frost, Lindberg, and Finer [21–23]. Multiple choice questions to be included in this construct include: 1) “How much time should a woman wait between giving birth and becoming pregnant again?” 2) “For the health of the mother and the baby, how much time should a woman wait between a miscarriage (a pregnancy that did not result in a birth) and becoming pregnancy again?” Response options for these two questions included: 1) zero to six months, 2) six months to a year, 3) one to two years, 4) more than two years, 5) you don’t know how much time she should wait. True or false questions adapted from Frost, Lindberg, and Finer [22] include: 1) “Contraceptive pills are effective even if a woman misses taking them for two or three days in a row;” 2) “After a woman stops taking contraceptive pills, it’s possible for her to get pregnant right away;” 3) “An IUD cannot be felt by a woman’s husband during sex;” 4) “An IUD can get permanently stuck in a woman’s body;” 5) “Women using an injectable contraceptive must get an injection every three months;” 6) “Using an injectable contraceptive can cause a woman to never be able to have children again;” 7) “Implants or an IUD can be removed early if a woman wants to get pregnant;” 8) “If a woman wanted to secretly delay having her next child, an IUD or injectable contraceptive could be used without anyone in her family knowing;” 9) “It’s okay to use the same condom more than once;” 10) “LAM is an effective form of delaying pregnancy even after the woman’s menstrual bleeding has returned after giving birth;” 11) “From one menstrual period to the next, are there certain days when a woman is more likely to become pregnant again;” and 12) “After the birth of a child, a woman can become pregnant before her menstrual period has returned.” Each correct response was assigned one point, with the resulting summary score ranging from 0 to 14.

##### Attitudes towards family planning

This measure consists of two items adapted from Camber Collective’s tool for segmentation of family planning clients in Niger [24] that reflect attitudes towards and acceptability of spacing and limiting births. The items are as follows: 1) “I think it is acceptable for a young wife to wait two years after having her first child to become pregnant again;” and 2) “It is acceptable for a couple to try to limit the number of children they have.” Response options for both questions were agree and disagree with one point assigned to each “agree” response. Based on their responses to these two questions, participants will be assigned a score between 0 and 2 with a higher score representing attitudes more supportive of modern contraceptive method use.

##### Perceived community norms regarding contraceptive method use

Participants are asked two questions about their perceptions of community acceptance regarding modern contraceptive method use. These items were developed to align with RMA intervention activities. The items are as follows: 1) “Men in your community think that it is acceptable for their wives to use a family planning method to delay or space pregnancies.” and 2) “People in your community think a young wife who uses a family planning method to delay or space pregnancies is not fulfilling her duty to her family.” Response options for both questions were agree and disagree with one point assigned to each “agree” response. Based on their responses, participants will each be assigned a score between 0 and 2 with a higher score representing perceived community norms more supportive of modern contraceptive method use.

##### Self-efficacy to use contraception

Participants are asked six questions to assess their self-efficacy, i.e., confidence in their ability to obtain, negotiate, and effectively use contraceptive methods. Items were adapted from recent research on this topic [25–27]. The six items are as follows: 1) “I feel confident in my ability to suggest to my husband that we wait a healthy amount of time to have another baby”, 2) “I feel confident in my ability to suggest to my husband that we use a family planning method,” 3) “I feel confident in my ability to persuade my husband to allow me to use a family planning method,” 4) “I could not continue to use a family planning method if I thought that my in-laws might find out” (reverse coded), 5) “If I wanted to use a family planning method to delay getting pregnant, I feel confident in my ability to be able to get the family planning method of my choice,” 6) “If I wanted to use a family planning method to delay getting pregnant, I feel confident in my ability to be able to use the family planning method of my choice correctly.” Response options were agree (point value = 1) and disagree (point value = 0). A summary score will be created, adding together participants’ responses and assigning participants a score of 0–6, with a higher score representing a higher level of self-efficacy.

##### Intention to use a modern contraceptive method

Intent to use a modern contraceptive method in the next three months will be assessed via a subset of the following four survey questions developed to align with RMA program goals and activities: 1) (for those currently using a modern contraceptive method - IUD, injectable, implant, pill, male condom, female condom, emergency contraception, LAM) “Will you continue to use [Current Method] over the next three months to avoid or delay pregnancy?” 2) (for those not currently using a modern contraceptive method) “Will you use a family planning method in the next three months to avoid or delay pregnancy?” 3) (for those who are currently pregnant) “After your current pregnancy is over, will you use a family planning method to space or delay pregnancy?” and 4) (if ‘yes’ to of any of the above) “Which family planning method would you prefer to use?” If participants respond that they intend to continue use for the next three months, start use in the next three months, or use after current pregnancy, and that the method that they prefer to use is considered a modern (vs. traditional) method, they will be considered as intending to use a modern contraceptive method in the next three months.

#### Data analysis

##### Planned analytic approach

A difference-in-difference approach utilizing multi-level, mixed-effects logistic regression models will be used to evaluate associations between intervention exposure and the primary and secondary outcomes identified above. The equivalent linear regression models will also be examined for significant findings in order to more clearly interpret results. These models will take into account the multiple study arms, cluster randomization, and repeated measurements over time. All models will include time (baseline or follow-up), treatment arm, district, and select covariates as fixed effects as well as a time by treatment interaction term. Descriptive analyses to describe the frequency of demographics and behavioral and social constructs as well as bivariate analyses to assess differences in demographics by treatment group and by loss to follow-up will be conducted. Any characteristics identified as significantly associated with treatment or female loss to follow-up at the *p* < 0.20 threshold will be considered as potential covariates (fixed effects) in adjusted models assessing effects of the intervention. Collinearity of covariates will be assessed, and highly collinear covariates may be removed from final models.

Study analyses will take into account clustering within villages using nested random effects specifications due to repeated measurements over time of individuals nested within villages. Models to assess the role of male engagement will include a three-level predictor of male participation in either household visits or small groups and include only those arms that received that intervention activity. Outcome analyses will use an intent-to-treat approach; per-protocol analyses will be conducted secondarily. Missing data on demographics and birth history will be computed where alternate data is available (e.g. calculating age from date of birth if age is not provided); however, no missing data will be imputed. All scales to be used as outcomes will be assessed for internal reliability at baseline using exploratory factor analysis and Cronbach’s alpha. If scales are multifactorial or not internally reliable, they may be modified to more accurately reflect the intended construct and/or be presented as individual item outcomes. Analyses will be conducted using STATA version 14.2® (StataCorp LLC, 2015) [28] and SAS version 9.4® (SAS Institute Inc., 2018) [29] programming applications. Trial results will be presented to the Ministry of Health of the Republic of Niger then communicated via peer review journals and scholarly meetings as well as meetings with national/international stakeholders.

##### Baseline data

At baseline, 1314 husbands and 1315 adolescent wives were contacted to participate in the study. Of these, 1156 and 1157, respectively, met inclusion criteria, consented to participate, and provided survey data, resulting in a baseline participation rate of 88% for both males and females. Upon review, data from 51 husbands and 60 adolescent wives were removed as a result of being highly incomplete or with data quality concerns. This left 1105 husbands and 1097 adolescent wives in our analytic sample. However, the leaders of one village decided post-baseline to discontinue participation in the study, requiring that data from this village be removed, resulting in a final analytic sample of 1080 men and 1072 women.

#### Data safety and monitoring

All precautions are taken to ensure that no harm comes to participants and that all data collection procedures maintain participants’ privacy. During data collection, each participant is assigned a unique participant identifier. Any personally identifiable information is collected solely for the purpose of re-identification and re-contacting of participants for follow-up data collection. Such identifiers are stored in encrypted files, separately from the main data and will be destroyed after 40-month follow-up data collection is complete.

During data collection, survey data is backed-up daily by the in-country research partner. These data will be uploaded to a secure server weekly, from which researchers at UCSD will download de-identified data to password-protected files on UCSD servers and access data through a limited access shared drive. Data shared with program partners or other stakeholders does not include any personal identifiers. All research staff at UCSD and at the in-country research partner, OASIS, are trained on these protection procedures and instructed to maintain absolute confidentiality of participants.

## Discussion

This study aims to assess the effects of the RMA intervention on modern contraceptive use among married AGYW and their husbands in the Dosso region of Niger using a four-arm cluster randomized controlled design. Early marriage is highly prevalent in Niger contributing to the early and frequent childbearing that drives elevated adolescent fertility rates and overall fertility rates. High adolescent fertility and overall fertility are associated with multiple poor health and development outcomes, including high rates of maternal and neonatal mortality. The RMA intervention was designed to provide education about contraception and promote gender equity in order to increase demand for and uptake of modern contraceptive methods in this population. The three approaches tested in the current study are designed to engage married AGYW, their husbands, and their communities in social norms change regarding gender equity and FP practices, to increase the likelihood that any observed improvements are sustained. The results of the currently described evaluation study will be provided to the Niger Ministry of Health and other in-country stakeholders to inform decisions regarding whether this approach is appropriate for scaling at regional and national levels.

The results of this trial will also examine whether this type of intervention may promote gender equitable norms and attitudes, and reduce IPV, seen to be a factor in contraceptive use across multiple global contexts. Results from this study will also contribute to the knowledge base regarding the utility of male engagement as a strategy within community-level approaches to promote modern contraceptive method use. Such knowledge is critical to improving the health of married AGYW, a highly vulnerable population that is difficult to reach with traditional models of health service.

## Supplementary information

**Additional file 1: Appendix A.** Informed Consent Materials.

## Data Availability

Not applicable.

## Abbreviations

AGYW: Adolescent girls and young women
CS: Case de santé
CSI: Case de santé intégré
DHS: Demographic and Health Survey
FACOMs: Community dialogue facilitators
FP: Family planning
HTSP: Healthy timing and spacing of pregnancy
IPV: Intimate partner violence
IUD: Intrauterine device
LAM: Lactational amenorrhea method
RAs: Research assistants
RMA: Reaching married adolescents
UCSD: University of California San Diego
