# Supplementary material for: Protocol for cluster randomized evaluation of reaching married adolescents - a gender-synchronized intervention to increase modern contraceptive use among married adolescent girls and young women and their husbands in Niger
Source: Reprod Health. 2019 Dec 18;16:180. doi: 10.1186/s12978-019-0841-3 (PMC6921454; doi:10.1186/s12978-019-0841-3)
Supplement: Supplementary file 1 — Additional file 1: Appendix A. Informed Consent Materials. [file 12978_2019_841_MOESM1_ESM.docx]

**Appendix A. Informed Consent Materials**

I. Female Consent Form

University of California, San Diego

Consent to Act as a Research Subject

IMPACT au Niger: Reaching Married Adolescents (RMA)

Female Survey Participant Consent Form

***Who is conducting the study, why you have been asked to participate, how you were selected, and what is the approximate number of participants in the study?***

Dr. Silverman, Professor at the University of California, School of Medicine is leading a research study to find out more about family health. Initiative OASIS Niger (OASIS) is the local research partner that is collecting information from participants under the direction of UCSD. The study is part of a larger project being implemented in the Dosso region by Pathfinder International. You have been asked to participate in this study because you are a young woman who is married and is between the ages of 13 and 19 living in the Dosso, Loga, or Doutchi districts. There will be approximately 2440 participants in this study; including 1200 young wives and 1200 husbands and 40 other community members across the Dosso region.

***Why is this study being done?***

The purpose of this study is to understand more about what type of health programs help the most in improving the health of young married women and their children. In particular, our goals are to:

1. Test three different programs and understand which is more helpful in improving the use of modern methods that women can use to have healthy spacing between births.
2. Understand how the cost is different across the three programs and which is most cost-effective in increasing the use of modern methods for healthy birth spacing.
3. Understand if people from the villages that receive these programs find them acceptable and useful.

***What will happen to you in this study and which procedures are standard of care and which are experimental?***

If you agree to be in this study, the following will happen to you:

You will complete two 60 minute surveys with an interviewer; one right now and one in 16-18 months (about a year and a half from now). In total, you will be enrolled in this study about two years. Nothing will be required of you during the one and a half year period between the first survey and the second survey.

The survey will include questions about your age at marriage, family decision-making, fertility history, and the roles of men and women at home and in the community. The survey will also contain questions related to family planning, including use of contraception, attitudes and desire to use contraception, knowledge about contraception, access to contraception, decision-making and communication around contraception. There are some other sensitive questions asked in this survey, including questions about experiences of violence from your husband that you may have had. Even if you choose to participate in this survey, though, you may choose to not answer any question that you do not want to answer. All of your responses to the questions asked will be kept confidential; no one, including your husband, family, village chief, will be told what you said during the survey.

***What risks are associated with this study?***

Participation in this study may involve some added risks or discomforts. These include the following:

1. There is a small risk that the information you provide could become known to another person. To minimize this risk, surveys will be conducted in private locations where you feel comfortable to speak freely. When your information is saved, your name and identifying information will not be stored with your responses to the questions so that no one will know that you are the one that gave those responses. Only OASIS staff will have access to your identifying information that connects your name with this code and it will only be used to identify you for the follow-up survey in 1.5 years. This information will be destroyed after the study ends and your name or identifying information will never be used in a report or publication of the results of this study. Research records will be kept confidential to the extent allowed by law. Research records may be reviewed by the UCSD Institutional Review Board and the Niger Ministry of Health Ethics Committee.
2. Participants may feel discomfort or embarrassment while providing personal information on sensitive issues like sexual behavior, negative experiences, and contraceptive practices. To reduce these risks, a female staff member adequately trained in sensitivity, providing support, and data collection will conduct the survey with female participants. A private location where you feel comfortable will be used to conduct the survey to avoid others from hearing your resposnses. The staff member will pause before asking sensitive questions to ensure privacy and remind you that you may choose to not answer any question you do not want to answer.

3. While we shared with you a detailed list of what topics this survey will cover, your husband, family, and community members will not be told this same list. They will be told it is a survey about family health. Only other young married women like you who are asked to participate in the survey will know the full list of topics. Husbands who participate in the husband survey will be asked questions on similar topics, but will not be asked sensitive questions, like for example, experiences of violence or perpetrating violence. We are doing this for the safety of all female participants in this study, to reduce the risk that they might experience violence as a result of participating in this study. Therefore, you are being asked to help ensure your safety and that of other women in this study by not sharing with others in your family or village that these sensitive questions were asked.

Because this is a research study, there may also be some unknown risks that are currently unforeseeable. You will be informed of any significant new findings that may impact your decision to continue participation.

***What are the alternatives to participating in this study?***

The alternative to participating in this study is to simply not participate. You may still participate in any health programs that are offered to you or your village, regardless of your participation in the survey.

***What benefits can be reasonably expected?***

There may or may not be any direct benefit to you from participating in this study. Participants will be provided with information about the health services available in your community that they might not have otherwise been aware of. The investigator, however, may learn more about the health and wellbeing of young married women like you and how to provide programs that may improve their health as well as that of their children, and society may benefit from this knowledge.

***Can you choose to not participate or withdraw from the study without penalty or loss of benefits?***

Participation in research is entirely voluntary. You may refuse to participate or withdraw or refuse to answer specific questions in an interview or on a questionnaire at any time without penalty or loss of benefits to which you are entitled. Your participation in the study will be stopped at your request and any data that you provided will be destroyed at your request.

If you wish not to participate in the study but are concerned about what other people in your family or village might think if you do not, it will not be shared with them that you did not participate and, if desired, options can be provided to hide the fact that you did not participate in the survey (e.g., the staff person will remain in the private location with you and speak with you on non-research topics for 30 minutes). Please tell us if you would like to do so and we can talk about a plan.

You will be told if any important new information is found during the course of this study that may affect your wanting to continue.

***Can you be withdrawn from the study without your consent?***

The researcher may remove you from the study without your consent if the researcher feels it is in your best interest or the best interest of the study.

***Will you be compensated for participating in this study?***

You will receive no compensation for participating in this research.

***Are there any costs associated with participating in this study?***

There will be no cost to you for participating in this study.

***Who can you talk to if you have questions?***

A research assistant from OASIS has explained this study to you and answered your questions. If you have other questions or research-related problems, you may reach the Director of OASIS, Dr. Nouhou Abdoul Moumouni, at +227-91658700 or the Program Director of Pathfinder International-Niger, Fatouma Azilaya, at +227-91919616. .

You may call the Ministry of Health Ethics Committee of Niger to inquire about your rights as a research subject or to report research-related problems.

MINISTERE DE LA SANTE PUBLIQUE DU NIGER

BP 613, Niamey-Niger

TEL: 20726960

Email: daidrp@gmail.com

The Ministry of Health Ethics Committee for Niger comprises of a group of people like doctors, researchers, and staff at the Ministry of Health who work towards safeguarding the rights of the study participants like you who take part in research studies undertaken in Niger.

You may also call the Human Research Protections Program Office in the United States at +1-858-246-4777 for more information about this study, to inquire about your rights as a research subject, or to report research-related problems.

HUMAN RESEARCH PROTECTIONS PROGRAM OFFICE (UCSD)

University of California, San Diego

Human Research Protections Program

9500 Gilman Drive, Mail Code 0052

La Jolla, California, 92093-0052

Do not provide your fingerprint on this consent form unless you have had a chance to ask questions and have received satisfactory answers to all of your questions.

If you agree to participate, please provide your stamped fingerprint on the line.

***Your Consent***

Do you agree to participate?

- YES
- NO

II. Male Consent Form

University of California, San Diego

Consent to Act as a Research Subject

IMPACT au Niger: Reaching Married Adolescents (RMA)

Male Survey Participant Consent Form

***Who is conducting the study, why you have been asked to participate, how you were selected, and what is the approximate number of participants in the study?***

Dr. Silverman, Professor at the University of California, School of Medicine is conducting a research study to find out more about family health. Initiative OASIS Niger is the local research partner that is collecting information from participants. The study is part of a larger project being implemented in the Dosso region by Pathfinder International. You have been asked to participate in this study because you are living in the Dosso, Loga, or Doutchi districts, married to a young woman between the ages of 13-19 who is also being asked to participate in this study. There will be approximately 2440 participants in this study; including 1200 young wives and 1200 husbands and 40 other community members across the Dosso region.

***Why is this study being done?***

The purpose of this study is to understand more about what type of health programs help the most in improving the health of young married women and their children. In particular, our goals are to:

1. Test three different programs and understand which is more helpful in improving factors related to healthy spacing between births among women.
2. Understand how the cost is different across the three programs and which is most cost-effective in increasing factors related to healthy birth spacing among women.
3. Understand if people from the villages that receive these programs find them acceptable and useful.

***What will happen to you in this study and which procedures are standard of care and which are experimental?***

If you agree to be in this study, the following will happen to you:

You will complete two 60 minute surveys with an interviewer; one right now and one in 16-18 months (about a year and a half from now). In total, you will be enrolled in this study about two years. Nothing will be required of you during the one and a half year period between the first survey and the second survey.

The survey will include questions about you, your wives, and children, family decision-making, and the roles of men and women at home and in the community. The survey will also contain questions related to family planning, including use of contraception, attitudes and desire to use contraception, knowledge about contraception, decision-making and communication around contraception. All of your responses to the questions asked will be kept confidential; no one, including your wives, family, village chief, will be told what you said during the survey.

***What risks are associated with this study?***

Participation in this study may involve some added risks or discomforts. These include the following:

1. There is a small risk that the information you provide could become known to another person. To minimize this risk, surveys will be conducted in private locations where you feel comfortable to speak freely. When your information is saved, your name and identifying information will not be stored with your responses to the questions so that no one will know that you are the one that said those responses. Only OASIS staff will have access to your identifying information that connects your name with this code and it will only be used to identify you for the follow-up survey in 1.5 years. This information will be destroyed after the study ends and your name or identifying information will never be used in a report or publication of the results of this study. Research records will be kept confidential to the extent allowed by law. Research records may be reviewed by the UCSD Institutional Review Board, the University of Michigan Institutional Review Board, and the Niger Ministry of Health Ethics Committee.
2. Participants may feel discomfort or embarrassment while providing personal information on sensitive issues like health and contraceptive practices. To reduce these risks, a male staff member adequately trained in sensitivity, providing support, and data collection will conduct the survey with male participants. A private location where you feel comfortable will be used to conduct the survey to avoid others from hearing your resposnses. The staff member will pause before asking sensitive questions to ensure privacy and remind you that you may choose to not answer any question that you do not want to answer.

Because this is a research study, there may also be some unknown risks that are currently unforeseeable. You will be informed of any significant new findings that may impact your decision to continue participation.

***What are the alternatives to participating in this study?***

The alternative to participating in this study is to simply not participate. You may still participate in any health programs that are offered to you or your village, regardless of your participation in the survey.

***What benefits can be reasonably expected?***

There may or may not be any direct benefit to you from participating in this study. Participants will be provided with information about the health services available in your community that they might not have otherwise been aware of. The investigator, however, may learn more about the health and wellbeing of young married women and their husbands, and how to provide programs that may improve their health as well as that of their children, and society may benefit from this knowledge.

***Can you choose to not participate or withdraw from the study without penalty or loss of benefits?***

Participation in research is entirely voluntary. You may refuse to participate or withdraw or refuse to answer specific questions in an interview or on a questionnaire at any time without penalty or loss of benefits to which you are entitled. Your participation in the study will be stopped at your request and any data that you provided will be destroyed at your request.

If you wish not to participate in the study but are concerned about what other people in your family or village might think if you do not, it will not be shared with them that you did not participate and, if desired, options can be provided to hide the fact that you did not participate in the survey. Please tell us if you would like to do so and we can talk about a plan.

You will be told of any important new information found during the course of this study that may affect your wanting to continue.

***Can you be withdrawn from the study without your consent?***

The researcher may remove you from the study without your consent if the researcher feels it is in your best interest or the best interest of the study.

***Will you be compensated for participating in this study?***

You will receive no compensation for participating in this research.

***Are there any costs associated with participating in this study?***

There will be no cost to you for participating in this study.

***Who can you talk to if you have questions?***

A research assistant from OASIS has explained this study to you and answered your questions. If you have other questions or research-related problems, you may reach the Director of OASIS, Dr. Nouhou Abdoul Moumouni, at +227-91658700 or the Program Director of Pathfinder International-Niger, Fatouma Azilaya, at +227-91919616

You may call the Ministry of Health Ethics Committee of Niger to inquire about your rights as a research subject or to report research-related problems.

MINISTERE DE LA SANTE PUBLIQUE DU NIGER

BP 613, Niamey-Niger

TEL: 20726960

Email: daidrp@gmail.com

The Ministry of Health Ethics Committee for Niger comprises of a group of people like doctors, researchers, and staff at the Ministry of Health who work towards safeguarding the rights of the study participants like you who take part in research studies undertaken in Niger.

You may also call the Human Research Protections Program Office in the United States at +1-858-246-4777 for more information about this study, to inquire about your rights as a research subject, or to report research-related problems.

HUMAN RESEARCH PROTECTIONS PROGRAM OFFICE (UCSD)

University of California, San Diego

Human Research Protections Program

9500 Gilman Drive, Mail Code 0052

La Jolla, California, 92093-0052

Do not provide your fingerprint on this consent form unless you have had a chance to ask questions and have received satisfactory answers to all of your questions.

If you agree to participate, please provide your stamped fingerprint on the line.

***Your Signature and Consent***

You have received a copy of this consent document.

Do you agree to participate?

- YES
- NO
